# Relationship of Higher-level Functional Capacity With Long-term Mortality in Japanese Older People: NIPPON DATA90

**DOI:** 10.2188/jea.JE20210077

**Published:** 2023-03-05

**Authors:** Hideki Nagata, Katsuyuki Miura, Sachiko Tanaka, Aya Kadota, Takehito Hayakawa, Keiko Kondo, Akira Fujiyoshi, Naoyuki Takashima, Yoshikuni Kita, Akira Okayama, Tomonori Okamura, Hirotsugu Ueshima

**Affiliations:** 1 Center for Epidemiologic Research in Asia, Shiga University of Medical Science, Otsu, Japan; 2 Department of Rehabilitation, Japanese Red Cross Nagoya Daini Hospital, Nagoya, Japan; 3 Department of Public Health, Shiga University of Medical Science, Otsu, Japan; 4 Department of Medical Statistics, Shiga University of Medical Science, Otsu, Japan; 5 Research Center for Social Studies of Health and Community Ritsumeikan University, Kyoto, Japan; 6 Department of Hygiene, Wakayama Medical University, Wakayama, Japan; 7 Department of Medicine, Kindai University, Osaka, Japan; 8 Tsuruga Nursing University, Fukui, Japan; 9 Research Institute of Strategy of Prevention, Tokyo, Japan; 10 Department of Preventive Medicine and Public Health, Keio University, Tokyo, Japan

**Keywords:** older people, disease-specific, higher-level functional capacity, mortality, sex difference

## Abstract

**Background:**

Basic and instrumental activities of daily living (BADL and IADL, respectively) are known predictors of mortality. However, the relationship between higher-level functional capacity (HLFC) and mortality and related sex differences have rarely been investigated.

**Methods:**

A prospective population-based cohort study was conducted in 1,824 older residents (≥65 years) with independent BADL from 300 randomly selected areas in Japan from 1995, and the participants were followed up until 2010. Using the Cox proportional hazards model, the relationship between HLFC and mortality risk was investigated, with adjustment for possible confounders. HLFC was assessed using the Tokyo Metropolitan Institute of Gerontology Index of Competence. Baseline data were collected using a questionnaire or by home-visit interviews.

**Results:**

During an average 12.2-year follow-up, all-cause death was observed in 836 (45.8%) participants. Impaired HLFC was significantly associated with mortality (hazard ratio [HR] 1.37; 95% confidence interval [CI], 1.13–1.65). Lower social role was significantly associated with higher mortality risk in men (HR 1.38; 95% CI, 1.13–1.68). Lower IADL and intellectual activity were significantly associated with higher mortality risk in women (HR 1.50; 95% CI, 1.15–1.95 and HR 1.46; 95% CI, 1.19–1.79, respectively). The relationship between HLFC and mortality risk showed a similar tendency among cardiovascular diseases, stroke, cancer, and pneumonia.

**Conclusion:**

Impaired HLFC was associated with a high risk of all-cause mortality among community-dwelling older people with independent BADL. In particular, social role in men and IADL and intellectual activity in women were associated with long-term mortality risk.

## INTRODUCTION

Lawton defined and systematized seven stages of competence, from the most basic function to the highest, which are known as life maintenance, functional health, perception-cognition, physical self-maintenance (corresponding to basic activities of daily living [BADL]), instrumental self-maintenance (corresponding to instrumental activities of daily living [IADL]), effectance (activity emanating from the motivation to explore and is known as intellectual activity), and social behavior.^1^ The last three stages (IADL, intellectual activity, and social behavior) constitute higher-level functional capacity (HLFC). The characteristics of HLFC include not only physical function but also intellectual and social factors.

Previous studies indicated that BADL limitation is independently associated with high mortality.^2^^–^^6^ HLFC usually deteriorates before BADL^7^; therefore, HLFC may predict mortality risk among the older people with normal BADL. Among HLFC stages, previous studies reported the relationship between intellectual activity and stroke incidence^8^ and between social isolation and health-related behavior.^9^ Two studies have investigated the relationship between HLFC and long-term mortality risk; however, they investigated in a limited area with shorter follow-up period, and did not exclude people with impaired baseline BADL.^10^^,^^11^ Moreover, although the prognostic value of BADL disability on mortality was slightly stronger among men,^4^ sex difference in the relationship between HLFC and mortality risk has been rarely investigated.^12^

The purpose of the present study was to examine the relationship between HLFC and long-term mortality risk and its sex difference in a representative, general sample of older Japanese men and women with independent BADL.

## METHODS

### Study participants

NIPPON DATA90 (National Integrated Project for Prospective Observation of Non-communicable Disease And its Trends in the Aged 1990) was a prospective cohort study in which a total of 8,383 community residents from 300 randomly selected areas from all over Japan participated and were followed up until November 2010. The details of this cohort study have been previously reported.^13^^–^^15^

In NIPPON DATA90, participants were surveyed for their BADL and HLFC in 1995. Participants aged 65 years or older in 1995 were eligible for the survey. This survey was performed by a public health center in each area. In the present study, we set the data from 1995 as the baseline because in that year, the first survey of BADL and HLFC in this cohort was conducted.

**Figure 1.**
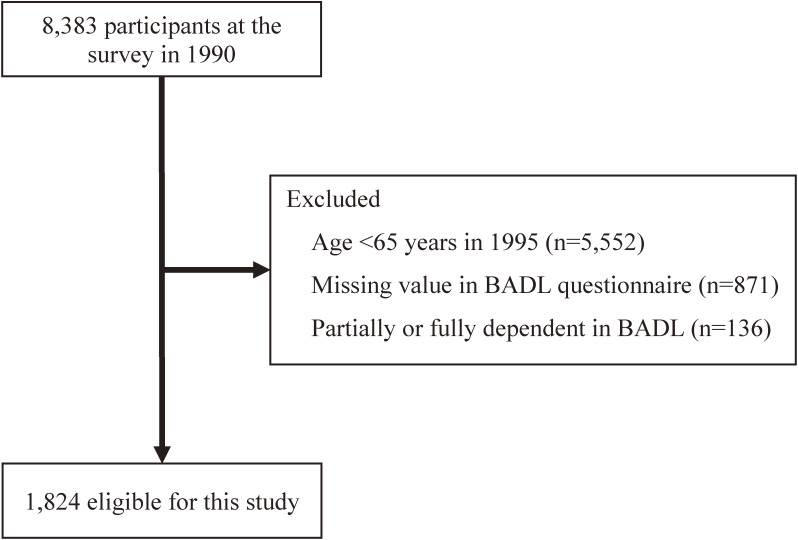
Flow diagram of study participants in the present analysis of the NIPPON DATA90. From 8,383 overall participants, we selected 1,824 participants after a total of 6,559 participants were excluded based on exclusion criteria. BADL, basic activities of daily living.

We used home-visit interviews to assess the participants; if this was impractical, the questions were asked over the phone or the questionnaire was mailed. A total of 6,559 participants were excluded for the following reasons: age <65 years (*n* = 5,552) in 1995, missing data in the BADL questionnaire (*n* = 871), and partially or fully dependent in BADL (*n* = 136). Accordingly, 1,824 participants (774 men and 1,050 women) were eligible for analysis (Figure 1). The present study was approved by the institutional Review Board of Shiga University of Medical Science (No. R2005-021).

### Baseline examinations

HLFC was evaluated at the 1995 survey using the Tokyo Metropolitan Institute of Gerontology Index of Competence (TMIG-IC). The TMIG-IC was developed to measure HLFC required to exceed BADL (based on Lawton’s hierarchical model of competence) specifically in older adults, with demonstrated reliability and validity,^16^ and the scale has been widely used.^8^^,^^15^^,^^16^ The TMIG-IC is a multidimensional 13-item index, and the response to each item is simply “yes” or “no” and is scored 1 for “yes” and 0 for “no.” The total score is the sum of the number of items answered “yes.” The TMIG-IC^16^ is presented in eTable 1. The maximum score of this index is 13 points, and a high score indicates normal HLFC. The participants whose total score was ≤9 were categorized as “impaired” HLFC and others, as “normal” HLFC, based on previous studies.^8^^,^^17^

The TMIG-IC consists of three subscales: IADL, intellectual activity, and social roles. Items 1 through 5 are classified as IADL, items 6 through 9 as intellectual activity, and items 10 through 13 as social roles. A participant was defined as being impaired if the score of the subscales was lower than the respective full score, ie, a participants with a score of ≤4 out of 5 for IADL or ≤3 out of 4 for intellectual activity or social roles was defined as “impaired” for subscales.^8^^,^^18^

The covariates were examined mainly from a survey in 1995 or 1990, if necessary. Age, sex, family members, past medical history, and BADL data were from the survey in 1995. Body mass index (BMI), smoking status, and alcohol intake were obtained from the survey in 1990. Detailed methods of the survey in 1990 have been described elsewhere.^13^^–^^15^ Family members were categorized into five groups: single, couple, two generations, three generations, and other. Past medical history was surveyed for stroke, myocardial infarction, and femoral fracture. The BADL contained six items: feeding, toileting, dressing, bathing, walking indoors, and walking outdoors. These items were graded as “independent”, “with partial help”, and “with full help”. BADL was regarded as “dependent” if at least one of the six items were performed “with partial help” or “with full help”. BMI was calculated as body weight (in kilograms) divided by the square of body height (in meters) and classified as <18.5 kg/m^2^, 18.5–24.9 kg/m^2^ or >24.9 kg/m^2^. Smoking status and alcohol intake were categorized into three groups: current, former, or never.

### Follow-up

The outcome of this study was defined as death due to all causes, cardiovascular diseases (CVD), cancer, and pneumonia during the follow-up period. We also set stroke deaths among CVD deaths as an outcome. The participants in this study were followed until November 2010. As previously reported,^13^ the survival status of the participants was followed using registration records required under the Family Registration Law in the municipalities where they resided. The causes of death in the National Vital Statistics were coded according to the 10^th^ International Classification of Disease (ICD-10). Deaths from CVD included ICD-10 codes I00–I99. Deaths from stroke included ICD-10 codes I60–69, from cancer included ICD-10 codes C00–D48, and from pneumonia included ICD-10 codes J12–18.

### Statistical analysis

Baseline characteristics were compared between participants with “normal” and “impaired” HLFC based on the total TMIG-IC score using the Chi-square test for categorical variables and *t*-test for continuous variables. The outcome for this present study was defined as death due to all causes, CVD, stroke, cancer, and pneumonia during the follow-up period.

Cox proportional hazards models were used to assess the relationship of functional capacity to these outcomes. First, we calculated the unadjusted hazard ratios (HRs) (model 1). HRs were adjusted for age and sex (model 2) and additionally adjusted for other confounding variables (BMI [three categories], smoking status [three categories], alcohol intake [three categories], family members [five categories], and past medical history) (model 3). After estimating HRs for total TMIG-IC, we calculated HRs for the subscales: IADL, intellectual activity, and social roles. All analyses were done in men and women, separately. Interactions by sex were assessed in all models and, if there was no significant interaction, analysis in total participants was also done. We also calculated the adjusted HR after excluding participants who died during the first 2 years from baseline examination to reduce reverse causality.

Results were represented as HRs and 95% confidence intervals (CIs) for each group. For all analyses, a two-tailed *P* value of <0.05 was considered statistically significant. All analyses were conducted using SAS version 9.4 (SAS Institute, Cary, NC, USA).

## RESULTS

During an average 12.2-year follow-up, all-cause death was observed in 836 (45.8%) participants. The mean age of the 1,824 participants was 72.5 years and 42.4% of the participants were men (Table 1). Of the 836 participants who died, 256, 270, 94, and 216 died because of CVD, cancer, pneumonia, and other reasons, respectively. Among those who died of CVD, stroke was the main cause in 95 participants. Crude all-cause mortality rates in total, impaired and normal HLFC people were 3,881, 8,673, and 3,360 per 100,000 person-years.

**Table 1.** Baseline characteristics, NIPPON DATA90, in 1990

	Total (*n* = 1,824)	HLFC^a^		*P* value^b^

		Normal (*n* = 1,593)	Impaired (*n* = 231)	
Age, years, mean (SD)	72.5 (5.9)	71.7 (5.3)	77.7 (7.2)	<0.001
Men, *n* (%)	774 (42.4)	687 (43.1)	87 (37.7)	0.116
Body mass index, *n* (%)				
<18.5 kg/m^2^	139 (7.6)	116 (7.3)	23 (10.0)	
18.5–24.9 kg/m^2^	1,222 (67.0)	1,061 (66.6)	161 (69.7)	
>24.9 kg/m^2^	463 (25.4)	416 (26.1)	47 (20.3)	0.090
Smoking status, *n* (%)				
Never	1,082 (59.3)	938 (58.9)	144 (62.3)	
Former	302 (16.6)	267 (16.8)	35 (15.2)	
Current	440 (24.1)	388 (24.4)	52 (22.5)	0.605
Alcohol intake, *n* (%)				
Never	1,291 (70.8)	1,123 (70.5)	168 (72.7)	
Former	91 (5.0)	79 (5.0)	12 (5.2)	
Current	442 (24.2)	391 (24.5)	51 (22.1)	0.715
Family member, *n* (%)				
Single	173 (9.5)	152 (9.5)	21 (9.1)	
Couple	540 (29.6)	499 (31.3)	41 (17.7)	
Two generations	429 (23.5)	365 (22.9)	64 (27.7)	
Three generations	606 (33.2)	515 (32.3)	91 (39.4)	
Other	76 (4.2)	62 (3.9)	14 (6.1)	<0.001
Past medical history, *n* (%)				
Stroke	94 (5.2)	74 (4.6)	20 (8.7)	0.036
Myocardial infarction	77 (4.2)	66 (4.1)	11 (4.8)	0.355
Femoral fracture	122 (6.7)	108 (6.8)	14 (6.1)	0.827

HLFC, higher-level functional capacity; SD, standard deviation; TMIG-IC, Tokyo Metropolitan Institute of Gerontology Index of Competence.

^a^HLFC was dichotomized into normal (TMIG-IC score = 10–13) and impaired (score = 0–9).

^b^Comparison between “Normal” and “Impaired” using *t*-test for continuous variables and Chi-square test for categorical variables.

Table 1 presents the baseline characteristics according to HLFC. Those with impaired HLFC based on total TMIG-IC score showed the following features: older age, low prevalence of living with a spouse, high prevalence of living with three generations, and high prevalence of stroke history, all at a significant level.

Table 2 shows the unadjusted and adjusted HRs and 95% CIs for the relationship between TMIG-IC score and all-cause mortality risk. The HRs generally decreased after adjustment for age (model 2), but not after adjustment for other potential confounding variables (model 3). Regarding the effect modification by sex, significant interactions were observed for IADL and intellectual activity (IADL, *P* = 0.008; intellectual activity, *P* = 0.004), but not for total TMIG-IC score or social role.

**Table 2.** Relationship of HLFC with all-cause mortality risk: 15-year follow-up of NIPPON DATA90, 1995–2010

Impaired domains	Men (*n* = 774)			Women (*n* = 1,050)			Total participants (*n* = 1,824)^a^		
		
	Model 1	Model 2	Model 3	Model 1	Model 2	Model 3	Model 1	Model 2	Model 3
	HR (95% CI)	HR (95% CI)	HR (95% CI)	HR (95% CI)	HR (95% CI)	HR (95% CI)	HR (95% CI)	HR (95% CI)	HR (95% CI)
Total TMIG-IC (impaired vs normal)									
	1.98	1.31	1.28	3.04	1.43	1.41	2.40	1.39	1.37
	(1.52–2.57)	(1.00–1.73)	(0.97–1.70)	(2.41–3.83)	(1.10–1.84)	(1.09–1.82)	(2.02–2.85)	(1.15–1.68)	(1.13–1.65)
IADL (impaired vs normal)									
	1.42	1.07	1.05	3.59	1.53	1.50	—	—	—
	(1.14–1.77)	(0.85–1.35)	(0.83–1.32)	(2.88–4.49)	(1.18–1.98)	(1.15–1.95)	—	—	—
Intellectual activity (impaired vs normal)									
	1.26	1.01	1.00	2.19	1.49	1.46	—	—	—
	(1.02–1.55)	(0.82–1.25)	(0.81–1.24)	(1.80–2.67)	(1.21–1.83)	(1.19–1.79)	—	—	—
Social role (impaired vs normal)									
	1.62	1.37	1.38	1.73	1.15	1.15	1.66	1.27	1.28
	(1.34–1.97)	(1.12–1.67)	(1.13–1.68)	(1.41–2.12)	(0.93–1.42)	(0.93–1.42)	(1.45–1.91)	(1.10–1.47)	(1.10–1.48)

CI, confidence interval; HLFC, higher-level functional capacity; HR, hazard ratio; IADL, instrumental activity of daily living; TMIG-IC, Tokyo Metropolitan Institute of Gerontology Index of Competence.

Model 1: unadjusted. Model 2: adjusted for age and sex (in total participants). Model 3: adjusted for age, sex, body mass index, smoking status, alcohol intake, family member, and past medical history.

HLFC was dichotomized into normal (Total TMIG-IC score = 10–13) and impaired (score = 0–9). IADL: normal (score = 5), impaired (score = 0–4). Intellectual activity: normal (score = 4), impaired (score = 0–3). Social role: normal (score = 4), impaired (score = 0–3).

^a^Results in total participants are shown, if there is no significant interactions between by sex.

In men, impaired social role showed a significantly increased risk of all-cause mortality, even after adjustment for potential confounding variables (HR 1.38; 95% CI, 1.13–1.68). However, no relationship was found for the total TMIG-IC score or the other two subscales (Table 2). In women, impairment of total TMIG-IC score, IADL, and intellectual activity was significantly associated with an elevated risk of all-cause mortality (HR 1.41; 95% CI, 1.09–1.82; HR 1.50; 95% CI, 1.15–1.95; and HR 1.46; 95% CI, 1.19–1.79, respectively). In total participants, those with impaired HLFC, based on total TMIG-IC score, had a significantly higher risk of all-cause death than those with normal HLFC, even after adjustment for potential confounding variables (HR 1.37; 95% CI, 1.13–1.65). Impaired social role also increased the risk of all-cause death.

The adjusted HRs of all-cause mortality after excluding deaths within 2 years from the baseline examination in total participants, men, and women were similar.

The unadjusted and adjusted HRs of mortality from CVD, stroke, cancer, and pneumonia are presented in eTable 2, eTable 3, eTable 4, and eTable 5, respectively. Regarding the effect modification by sex, a significant interaction were observed for IADL, but not for other scales. In men, impaired social role significantly increased the mortality risk from the four diseases, but not in women. In contrast, in women, impaired IADL and intellectual activity were significantly associated with a high mortality risk from the four diseases. However, in men, a significant relationship was not found for IADL and intellectual activity. The total TMIG-IC score was significantly associated with the risk of mortality from CVD, stroke, cancer, and pneumonia in both men and women and in total participants.

## DISCUSSION

In this long-term follow-up study of a representative, older sample of Japanese men and women, we found that (1) impaired HLFC increased the risk of all-cause mortality by 37% among the older people with independent BADL at baseline; (2) among the three subscales of HLFC, impaired social role increased the risk of all-cause mortality by 38% in men; (3) in women, impaired IADL and intellectual activity increased the risk of all-cause mortality by 50% and 46%, respectively; and (4) relationships between the three subscales and mortality risk from CVD, stroke, cancer, and pneumonia were similar.

We found that impaired HLFC was associated with a mortality risk. Those with impaired HLFC were older than those with normal HLFC and had a stroke history, suggesting that the effect of impaired HLFC on mortality risk was partially influenced by age and stroke history. However, the significance of the relationship between HLFC and mortality risk remained even after adjusting for age, stroke history, and other confounding variables. Although one study showed that impaired HLFC is associated with mortality,^10^ this study focused on the octogenarian population living in a limited area. Several previous studies have shown that impaired functional capacity is associated with mortality^2^^–^^6^; however, these studies focused mainly on lower-level functional capacity, such as BADL, and did not examine HLFC required to exceed BADL. This may be because an appropriate scale has not been constructed for measuring HLFC in older adults. Another study also reported the importance of HLFC for mortality^11^; however, the participants were recruited in a limited area, the follow-up period was shorter, and the study did not exclude participants dependent in BADL. Our study participants were from a representative Japanese sample with independent BADL. Thus, the results would be more generalizable for older adults. An assessment of HLFC may enable early detection of older adults who are at an increased risk of mortality and possible preventive interventions.

We also found significant interactions by sex. In men, only social role was significantly associated with mortality risk. The social role subscale includes four items related to social relationships. The World Health Organization proposed the importance of promoting HLFC, especially social participation, and not just the ability to be physically active or participate in the labor force, in its policy of “Active Aging”.^19^ Men who engaged in social participation, such as eating with others, had better dietary quality, higher vegetable intake, and better mental health than women.^20^ This study showed that eating with others was an independent survival factor only in men. In general, it has been recognized that social relationships influence mortality among men and women.^21^ A previous study in Japan reported that friendships among women focus on intimacy and disclosure, whereas men are oriented toward specific activities.^22^ Therefore, men may derive greater health benefits from social participation than women.^22^ It means that the lack of social participation may increase mortality risk in men but not in women. Our results showed a positive effect of sufficient social activity on the mortality risk in men. Maintaining a good social role may prevent the onset of diseases and decrease the mortality risk in community-dwelling older men.

In contrast, IADL and intellectual activity were significantly associated with mortality only in women. These results were inconsistent with those of previous studies, which showed that IADL limitation was associated with mortality in men and women, but these studies did not exclude people with impaired BADL.^2^^–^^6^ BADL is impaired by chronic diseases, such as stroke history, severe heart failure, chronic obstructive pulmonary disease, and severe injury. We excluded people with impaired BADL in the present study because the purpose of the present study was focused on the ability to exceed BADL.

IADL includes activities, such as working around the house, accessing transportation, preparing meals, and managing money independently. Thus, IADL may have been influenced not only by physical and cognitive function but also by lifestyle. A previous study in Japan demonstrated that men were less likely to cook than women.^23^ In addition, Japanese men are more likely to have a driver’s license than women.^24^ Especially in men, IADL score could be influenced by these factors. In the present study, IADL was associated with mortality only in women. However, some lifestyles, such as cooking and driving a car, should be considered in future studies. On the other hand, the relationship of intellectual activity to mortality was shown only in women, which is similar to a previous study showing that intellectual activity was significantly associated with stroke incidence only in women.^8^ The items of the intellectual activity subscale can be categorized as cognitively stimulating activities, which have been found to be protective for cognitive decline.^25^ Frequent participation in cognitively stimulating activities (ie, reading newspapers, books, and magazines) may reduce mortality risk by retaining cognitive function. These factors may affect the gender-specific relationship of HLFC to mortality.

Another unique finding of the present study was that the relationships of total TMIG-IC score and its three subscales with mortality risk were similar among the four diseases (ie, CVD, stroke, cancer, and pneumonia). The three subscales were related to going outside, understanding information, making decisions, and obtaining information. These functional abilities are needed to promote health literacy and health behavior. Previous studies have shown that HLFC is associated with stroke risk^8^ and that socially isolated people are less likely to have good health behaviors than non-isolated people.^9^ Such findings may explain the relationship between HLFC and disease-specific mortality risk in our study.

This study has several limitations. First, in the present study, socioeconomic factors were not examined. It is possible that socioeconomic factors were upper streams of HLFC and affected the causal relationship between HLFC and mortality risk. Second, other conventional risk factors of the diseases, such as cognitive function and psychological status, were not examined in our study. BADL, IADL, intellectual activity, and social role could be influenced by cognitive function. If we could consider cognitive function in the present study, the relationship between HLFC and mortality might be different. However, one of the strengths of this study is that we excluded participants with impaired BADL, which could be associated with cognitive decline. This exclusion could minimize the confounding effect by cognitive decline. Third, data on BMI, smoking status, and alcohol intake data were taken from the survey in 1990. These conditions might have changed from those at the baseline in 1995. Finally, the validity and reliability of the TMIG-IC have been confirmed only in the Japanese population.^16^ However, this index is based on Lawton’s systematized seven stages of competence from the basic to the highest function,^1^ so the concept of TMIG-IG could be applied in other countries.

In conclusion, this study provides new and important information about the relationship between HLFC and mortality risk and the related sex differences. The mortality risk increased among those with impaired HLFC at baseline. In particular, engagement in social activity, in the case of men, and IADL and intellectual activity, in the case of women, were significantly associated with mortality risk. Monitoring the HLFC in the older people might be useful for identifying those at a high risk of death.

## SUPPLEMENTARY MATERIAL

eTable 1. Tokyo Metropolitan Institute of Gerontology Index of Competence

eTable 2. Relationship of HLFC with CVD mortality risk: 15-year follow-up of NIPPON DATA90, 1995–2010

eTable 3. Relationship of HLFC with stroke mortality risk: 15-year follow-up of NIPPON DATA90, 1995–2010

eTable 4. Relationship of HLFC with cancer mortality risk: 15-year follow-up of NIPPON DATA90, 1995–2010

eTable 5. Relationship of HLFC with pneumonia mortality risk: 15-year follow-up of NIPPON DATA90, 1995–2010
