# Supplementary material for: Relationship of Higher-level Functional Capacity With Long-term Mortality in Japanese Older People: NIPPON DATA90
Source: J Epidemiol. 2023 Mar 5;33(3):136–41. doi: 10.2188/jea.JE20210077 (PMC9909170; doi:10.2188/jea.JE20210077)
Supplement: Supplementary file 1 [file je-33-136-s001.pdf]

eTable 1. Tokyo Metropolitan Institute of Gerontology Index of Competence

Instrumental Activities of Daily Living (IADL)			
1	Can you use public transportation (bus or train) by yourself?	1. Yes	0. No
2	Are you able to shop for daily necessities?	1. Yes	0. No
3	Are you able to prepare meals by yourself?	1. Yes	0. No
4	Are you able to pay bills?	1. Yes	0. No
5	Can you handle your own banking?	1. Yes	0. No
Intellectual Activity			
6	Are you able to fill out forms for your pension?	1. Yes	0. No
7	Do you read newspapers?	1. Yes	0. No
8	Do you read books or magazines?	1. Yes	0. No
9	Are you interested in news stories or programs dealing with health?	1. Yes	0. No
Social Role			
10	Do you visit the homes of friends?	1. Yes	0. No
11	Are you sometimes called on for advice?	1. Yes	0. No
12	Are you able to visit sick friends?	1. Yes	0. No
13	Do you sometimes initiate conversations with young people?	1. Yes	0. No

The maximum score of this index is 13 points, and a high score indicates normal higher-level functional capacity.

eTable 2. Relationship of HLFC with CVD mortality risk: 15-year follow-up of NIPPON DATA90, 1995–2010

Impaired domains	Men (n=774)			Women (n=1,050)			Total participants (n=1,824)[a]		
	Model 1 HR (95% CI)	Model 2 HR (95% CI)	Model 3 HR (95% CI)	Model 1 HR (95% CI)	Model 2 HR (95% CI)	Model 3 HR (95% CI)	Model 1 HR (95% CI)	Model 2 HR (95% CI)	Model 3 HR (95% CI)
Total TMIG-IC (impaired vs. normal)									
	1.62 (1.27–2.08)	1.37 (1.07–1.76)	1.36 (1.05–1.75)	1.67 (1.37–2.03)	1.34 (1.09–1.65)	1.33 (1.08–1.64)	1.62 (1.39–1.89)	1.34 (1.14–1.57)	1.33 (1.13–1.56)
IADL (impaired vs. normal)									
	1.24 (1.03–1.50)	1.08 (0.89–1.31)	1.09 (0.89–1.33)	1.83 (1.51–2.23)	1.40 (1.14–1.73)	1.40 (1.13–1.73)	– –	– –	– –
Intellectual activity (impaired vs. normal)									
	1.11 (0.93–1.32)	0.99 (0.82–1.18)	0.98 (0.82–1.18)	1.37 (1.19–1.57)	1.23 (1.07–1.41)	1.23 (1.07–1.42)	1.23 (1.10–1.37)	1.12 (1.00–1.25)	1.12 (1.00–1.25)
Social role (impaired vs. normal)									
	1.30 (1.10–1.53)	1.22 (1.03–1.45)	1.24 (1.04–1.47)	1.25 (1.08–1.43)	1.13 (0.98–1.30)	1.13 (0.98–1.30)	1.26 (1.13–1.41)	1.17 (1.04–1.30)	1.17 (1.05–1.30)

CI, confidence interval; HLFC, higher-level functional capacity; HR, hazard ratio; IADL, instrumental activity of daily living; TMIG-IC, Tokyo Metropolitan Institute of Gerontology Index of Competence.

Model 1: unadjusted. Model 2: adjusted for age and sex (in total participants). Model 3: adjusted for age, sex, body mass index, smoking status, alcohol intake, family member, and past medical history.

HLFC was dichotomized into normal (Total TMIG-IC score=10–13) and impaired (score=0–9). IADL: normal (score=5), impaired (score=0–4). Intellectual activity: normal (score=4), impaired (score=0–3). Social role: normal (score=4), impaired (score=0–3).

[a] Results in total participants are shown, if there is no significant interactions between by sex.

eTable 3. Relationship of HLFC with stroke mortality risk: 15-year follow-up of NIPPON DATA90, 1995–2010

Impaired domains	Men (n=774)			Women (n=1,050)			Total participants (n=1,824)[a]		
	Model 1 HR (95% CI)	Model 2 HR (95% CI)	Model 3 HR (95% CI)	Model 1 HR (95% CI)	Model 2 HR (95% CI)	Model 3 HR (95% CI)	Model 1 HR (95% CI)	Model 2 HR (95% CI)	Model 3 HR (95% CI)
Total TMIG-IC (impaired vs. normal)									
	1.64	1.35	1.33	1.80	1.38	1.36	1.71	1.35	1.34
	(1.30–2.07)	(1.06–1.71)	(1.05–1.69)	(1.50–2.16)	(1.14–1.67)	(1.12–1.65)	(1.48–1.97)	(1.16–1.57)	(1.15–1.56)
IADL (impaired vs. normal)									
	1.26	1.08	1.07	2.06	1.50	1.49	–	–	–
	(1.06–1.51)	(0.90–1.29)	(0.89–1.29)	(1.72–2.46)	(1.23–1.82)	(1.22–1.81)	–	–	–
Intellectual activity (impaired vs. normal)									
	1.15	1.01	1.01	1.38	1.21	1.21	1.25	1.12	1.11
	(0.97–1.35)	(0.85–1.20)	(0.85–1.19)	(1.21–1.57)	(1.06–1.38)	(1.06–1.38)	(1.13–1.38)	(1.01–1.24)	(1.00–1.24)
Social role (impaired vs. normal)									
	1.33	1.24	1.24	1.27	1.12	1.12	1.29	1.17	1.17
	(1.13–1.55)	(1.06–1.45)	(1.06–1.46)	(1.11–1.45)	(0.98–1.28)	(0.98–1.29)	(1.17–1.43)	(1.05–1.30)	(1.06–1.30)

CI, confidence interval; HLFC, higher-level functional capacity; HR, hazard ratio; IADL, instrumental activity of daily living; TMIG-IC, Tokyo Metropolitan Institute of Gerontology Index of Competence.

Model 1: unadjusted. Model 2: adjusted for age and sex (in total participants). Model 3: adjusted for age, sex, body mass index, smoking status, alcohol intake, family member, and past medical history.

HLFC was dichotomized into normal (Total TMIG-IC score=10–13) and impaired (score=0–9). IADL: normal (score=5), impaired (score=0–4). Intellectual activity: normal (score=4), impaired (score=0–3). Social role: normal (score=4), impaired (score=0–3).

[a] Results in total participants are shown, if there is no significant interactions between by sex.

eTable 4. Relationship of HLFC with cancer mortality risk: 15-year follow-up of NIPPON DATA90, 1995–2010

Impaired domains	Men (n=774)			Women (n=1,050)			Total participants (n=1,824)[a]		
	Model 1 HR (95% CI)	Model 2 HR (95% CI)	Model 3 HR (95% CI)	Model 1 HR (95% CI)	Model 2 HR (95% CI)	Model 3 HR (95% CI)	Model 1 HR (95% CI)	Model 2 HR (95% CI)	Model 3 HR (95% CI)
Total TMIG-IC (impaired vs. normal)									
	1.85 (1.45–2.36)	1.52 (1.18–1.95)	1.48 (1.15–1.90)	1.76 (1.46–2.13)	1.36 (1.12–1.66)	1.35 (1.11–1.65)	1.77 (1.53–2.06)	1.40 (1.20–1.63)	1.39 (1.19–1.62)
IADL (impaired vs. normal)									
	1.31 (1.08–1.59)	1.10 (0.90–1.34)	1.08 (0.88–1.33)	2.14 (1.78–2.57)	1.59 (1.30–1.94)	1.59 (1.30–1.94)	– –	– –	– –
Intellectual activity (impaired vs. normal)									
	1.30 (1.09–1.55)	1.13 (0.94–1.35)	1.13 (0.94–1.36)	1.35 (1.18–1.55)	1.20 (1.04–1.37)	1.19 (1.04–1.37)	1.31 (1.18–1.46)	1.16 (1.04–1.29)	1.16 (1.04–1.29)
Social role (impaired vs. normal)									
	1.44 (1.22–1.71)	1.35 (1.13–1.60)	1.37 (1.15–1.63)	1.20 (1.04–1.38)	1.06 (0.92–1.22)	1.06 (0.92–1.23)	1.29 (1.16–1.43)	1.16 (1.04–1.30)	1.17 (1.05–1.31)

CI, confidence interval; HLFC, higher-level functional capacity; HR, hazard ratio; IADL, instrumental activity of daily living; TMIG-IC, Tokyo Metropolitan Institute of Gerontology Index of Competence.

Model 1: unadjusted. Model 2: adjusted for age and sex (in total participants). Model 3: adjusted for age, sex, body mass index, smoking status, alcohol intake, family member, and past medical history.

HLFC was dichotomized into normal (Total TMIG-IC score=10–13) and impaired (score=0–9). IADL: normal (score=5), impaired (score=0–4). Intellectual activity: normal (score=4), impaired (score=0–3). Social role: normal (score=4), impaired (score=0–3).

[a] Results in total participants are shown, if there is no significant interactions between by sex.

eTable 5. Relationship of HLFC with pneumonia mortality risk: 15-year follow-up of NIPPON DATA90, 1995–2010

Impaired domains	Men (n=774)			Women (n=1,050)			Total participants (n=1,824)[a]		
	Model 1 HR (95% CI)	Model 2 HR (95% CI)	Model 3 HR (95% CI)	Model 1 HR (95% CI)	Model 2 HR (95% CI)	Model 3 HR (95% CI)	Model 1 HR (95% CI)	Model 2 HR (95% CI)	Model 3 HR (95% CI)
Total TMIG-IC (impaired vs. normal)									
	1.55 (1.22–1.97)	1.31 (1.03–1.68)	1.28 (1.00–1.64)	1.65 (1.37–1.99)	1.26 (1.04–1.53)	1.25 (1.02–1.52)	1.59 (1.37–1.84)	1.27 (1.09–1.48)	1.26 (1.08–1.46)
IADL (impaired vs. normal)									
	1.19 (0.99–1.43)	1.04 (0.86–1.25)	1.02 (0.84–1.24)	1.94 (1.62–2.32)	1.41 (1.16–1.71)	1.40 (1.15–1.71)	– –	– –	– –
Intellectual activity (impaired vs. normal)									
	1.15 (0.98–1.36)	1.03 (0.87–1.23)	1.02 (0.86–1.21)	1.33 (1.17–1.52)	1.17 (1.02–1.34)	1.17 (1.02–1.33)	1.23 (1.11–1.36)	1.11 (1.00–1.23)	1.10 (0.99–1.22)
Social role (impaired vs. normal)									
	1.32 (1.13–1.55)	1.25 (1.07–1.47)	1.26 (1.07–1.48)	1.22 (1.06–1.39)	1.07 (0.94–1.23)	1.08 (0.94–1.23)	1.26 (1.13–1.39)	1.14 (1.03–1.27)	1.15 (1.04–1.28)

CI, confidence interval; HLFC, higher-level functional capacity; HR, hazard ratio; IADL, instrumental activity of daily living; TMIG-IC, Tokyo Metropolitan Institute of Gerontology Index of Competence.

Model 1: unadjusted. Model 2: adjusted for age and sex (in total participants). Model 3: adjusted for age, sex, body mass index, smoking status, alcohol intake, family member, and past medical history.

HLFC was dichotomized into normal (Total TMIG-IC score=10–13) and impaired (score=0–9). IADL: normal (score=5), impaired (score=0–4). Intellectual activity: normal (score=4), impaired (score=0–3). Social role: normal (score=4), impaired (score=0–3).

[a] Results in total participants are shown, if there is no significant interactions between by sex.
